# Impact of Social Processes in Online Health Communities on Patient Empowerment in Relationship With the Physician: Emergence of Functional and Dysfunctional Empowerment

**DOI:** 10.2196/jmir.7002

**Published:** 2017-03-13

**Authors:** Gregor Petrič, Sara Atanasova, Tanja Kamin

**Affiliations:** ^1^ Centre for Methodology and Informatics Faculty of Social Sciences University of Ljubljana Ljubljana Slovenia; ^2^ Centre for Social Psychology Faculty of Social Sciences University of Ljubljana Ljubljana Slovenia

**Keywords:** physician-patient relations, patient empowerment, patient compliance, conflict, online health community, eHealth literacy, cross-sectional survey

## Abstract

**Background:**

Substantial research demonstrates the importance of online health communities (OHCs) for patient empowerment, although the impact on the patient-physician relationship is understudied. Patient empowerment also occurs in relationship with the physician, but studies of OHCs mostly disregard this. The question also remains about the nature and consequences of this empowerment, as it might be based on the limited validity of some information in OHCs.

**Objective:**

The main purpose of this study was to examine the impact of social processes in OHCs (information exchange with users and health professional moderators, social support, finding meaning, and self-expressing) on functional and dysfunctional patient empowerment in relationship with the physician (PERP). This impact was investigated by taking into account moderating role of eHealth literacy and physician’s paternalism.

**Method:**

An email list–based Web survey on a simple random sample of 25,000 registered users of the most popular general OHC in Slovenia was conducted. A total of 1572 respondents completed the survey. The analyses were conducted on a subsample of 591 regular users, who had visited a physician at least once in the past 2 years. To estimate the impact of social processes in OHC on functional and dysfunctional PERP, we performed a series of hierarchical regression analyses. To determine the moderating role of eHealth literacy and the perceived physician characteristics, interactions were included in the regression analyses.

**Results:**

The mean age of the respondents in the sample was 37.6 years (SD 10.3) and 83.3% were females. Factor analyses of the PERP revealed a five-factor structure with acceptable fit (root-mean-square error of approximation =.06). Most important results are that functional self-efficacy is positively predicted by information exchange with health professional moderators (beta=.12, *P*=.02), information exchange with users (beta=.12, *P*=.05), and giving social support (beta=.13, *P*=.02), but negatively predicted with receiving social support (beta=−.21, *P*<.001). Functional control is also predicted by information exchange with health professional moderators (beta=.16, *P*=.005). Dysfunctional control and competence are inhibited by information exchanges with health professionals (beta=−.12, *P*=.03), whereas dysfunctional self-efficacy is inhibited by self-expressing (beta=−.12, *P*=.05). The process of finding meaning likely leads to the development of dysfunctional competences and control if the physician is perceived to be paternalistic (beta=.14, *P*=.03). Under the condition of high eHealth literacy, the process of finding meaning will inhibit the development of dysfunctional competences and control (beta=−.17, *P*=.01).

**Conclusions:**

Social processes in OHCs do not have a uniform impact on PERP. This impact is moderated by eHealth literacy and physician paternalism. Exchanging information with health professional moderators in OHCs is the most important factor for stimulating functional PERP as well as diminishing dysfunctional PERP. Social support in OHCs plays an ambiguous role, often making patients behave in a strategic, uncooperative way toward physicians.

## Introduction

### State of Research on Role of Online Health Communities in Patient Empowerment in Relationship With the Physician

Online health communities (OHCs), one of the most important eHealth Internet applications [1], play an important role in the process of transformation from traditional physician-centered relationships to patient-centered relationships [2,3]. This process is often hailed as the emergence of the empowered patient [4,5]. OHCs such as PatientsLikeMe, WebMD, and MedHelp are environments that can provide more relevant health information than search engines [6]. OHCs allow users to share health-related experiences, exchange social support, compare information on health issues, find meaning, and have discussions with online health professional moderators who provide health consultations and clinical expertise to the users [7-11]. These processes lead to individual and collective empowerment [10,12,13] and may consequently change the dynamics of patient-physician relationships [4,13-15]. In OHCs, users can engage in physician previsit activities, such as reading posts and asking questions [16]. There exists an association among exchanging social support, comparing with others, sharing experiences and expressing greater confidence in relationships with physicians [4,13,15]. In one study, 60.3% of active users reported that their use of OHCs affected their relationships with their physicians [4].

Although the above-mentioned studies provide us valuable information regarding the impacts of OHCs on patient empowerment in relationship with the physician (PERP), little is known about how some potentially problematic facets of patient empowerment in OHCs [6,17,18] are associated with patient’s relationship with the physician. Even though concerns have been expressed about access to misinformation and patients’ inability to understand, evaluate and process relevant information under conditions of low eHealth literacy [19], studies have shown only positive effects on patient-physician relationship from using OHCs. This is due to the fact that previous studies understand PERP as a somewhat narrow concept and measure it mostly with single items. Being prepared for the visit [16] and having the intention to actively communicate with a physician [4,15] are important facets of PERP; however, this concept needs to be expanded to more systematic and thorough observation of the positive and negative facets of participation, control, confidence, efficacy, and patient skills in this relationship. In addition, the following factors need to be considered in the scope of the concept such as patient empowerment does not necessarily lead to better communication with the physician [20], has limitations [5], can be unproductive for the relationship, can lead to negative encounters [6,21] and nonadherence [18], may induce conflicts, and can enable manipulations and even revenge [22,23].

### Functional and Dysfunctional Empowerment of Patient in Relationship With Physician

In this study, we propose that the positive and negative aspects of PERP can be systematically conceptualized by leaning on the theory of psychological empowerment by Zimmerman [24] and the distinction between communicative and strategic orientation toward partner in interaction by Habermas [25]. In general, we can differentiate between two types of patient empowerment. One focuses on the individual patient and his or her personal transformation, whereas the other occurs in the context of the patient-physician relationship [26]. This distinction can be observed in many conceptualizations and measurements of patient empowerment, but is usually not manifested as such [27]. Zimmerman dimensions of psychological empowerment (gaining self-efficacy, sense of control, and competences) were understood in subsequent research exclusively as individual characteristics, yet he claimed that they also refer to qualities of social interaction and communication [24]. Therefore, we propose that these components can also be applied at the level of communicative behavior of the patient in relation to his or her physician. We can synthesize that self-efficacy refers to a patient’s confidence and ability to achieve his or her goals in an encounter with the physician and patient’s awareness of the outcomes he or she wants from the interaction [28]. Patient’s control in a relationship with the physician signifies the intention and ability to participate in shared decision making [26,27,29] and the development of tailored treatment plans [30]. Competences pertain to a self-assessed mastery in accomplishing tasks and coping with role-related situations [24,31], as well as gaining the skills and abilities needed to have a meaningful discussion with a physician [7,15,30,32].

Therefore, PERP is a form of communicative orientation and behavior that is an important determinant of a relationship with the physician and of high-quality health care [20,33]. This communicative behavior, however, is not always positive or productive for the relationship, as it is often reminded that changing power positions and associated processes can result in the emergence of conflicts between a patient and his or her physician [9,19,34]. This can result in patient-physician distrust, the patient being perceived as difficult, the patient becoming overconfident, or the patient provoking and taking an aggressive stance toward the physician [22,23,35,36]. We suggest that there are essentially two different forms of orientation toward the physician, which can result in PERP that can have functional or dysfunctional consequences.

This assumption is based on the main premise of one of the most influential sociological theories: the theory of communicative action [25]. This theory suggests that a person in an intersubjective situation can generally relate to other person in a strategic or a communicative way. A strategic orientation pursues maximizing the effectiveness of influencing the actions, decisions, and expectations of other involved in an interaction. Conversely, a communicative orientation denotes social actions in which actors coordinate themselves and attain their goals based on mutual efforts to achieve understanding [25]. In a strategic orientation, patient acts exclusively toward his or her own gains and considers the physician as means for accomplishing his or her own goals. Such a patient does not acknowledge the interests of the physician, which results in interactions that can be misleading, insincere, disrespectful, and dominating. The power that patient gains from strategic (inter)action is manifested in forms of domination, force, and coercion and is thus an illegitimate power with dysfunctional consequences for the relationship [12]. In contrast, the power emanating from communicative (inter)action can be described as a legitimate form of power that is directed at reaching agreement.

**Table 1:** Components of patient empowerment in a relationship with the physician (PERP).

Orientation to physician	Self-efficacy	Control	Competence
Communicative orientation	Prepared to have a collaborative and efficient encounter; aims for efficient communication; is able to attract attention	Has the confidence to make propositions and express doubts; willing to communicate information; willing to participate and decide	Is able to understand physician; is able to retain and repeat information; is able to describe symptoms
Strategic orientation	Deceives physician with intention of getting desired medicine, getting more sick leave than needed or simulating disease	Expresses aggression and hostility; disregards advice; intentionally undermines authority	Provokes with information; exhibits overconfidence; disregards professional knowledge; assumes authoritative role

If we merge Zimmerman dimensions of empowerment and the two types of patient orientations, we arrive at the typology of 6 components of the PERP, of which 3 are forms of functional empowerment and 3 are forms of dysfunctional empowerment (Table 1). A functional PERP appears when patients exert self-efficacy, control, and competences in relationship with the physician based on a communicative orientation, which will likely lead to collaborative, mutual, and open communication that is beneficial for all the parties involved [33,37]. Empowerment in such cases enables a productive partnership with a physician that leads to positive health outcomes [4]. Conversely, a dysfunctional PERP appears when self-efficacy, control, and competences are exerted in a strategic way in which the physician is no more than a means to the patient’s end [38]. This opens the possibilities for the patient to perform manipulative tasks to obtain the treatment that he or she wants without negotiating with the physician such as (1) to exert anger and rejection [39], (2) to intentionally undermine authority [40], (3) to simulate disease, or (4) to assume an authoritative role [5]. A dysfunctional PERP is characterized by the patient’s strategic orientation, which may lead to disruptions in the relationship with the physician and even its breakdown. It may also lead eventually to severe, problematic health outcomes for the patient.

### Aim of Research

The main goal of this study was to investigate how social processes, in which patients are involved in OHCs (such as exchanging information with other users and health professional moderators, exchanging social support, finding meaning and recognition, self-expression), are associated with patient’s functional and dysfunctional PERP. An investigation of this association needs to consider the moderating role of dominant factors of the patient-physician relationship, such as the physician’s willingness to give up the paternalistic role [1,9,19] and the level of eHealth literacy, which limits patient empowerment and elicit conflicts with physicians [5]. The research questions of this study are as follows:

RQ_1_: What is the impact of social processes in OHCs on functional and dysfunctional PERP?

RQ_2_: Do eHealth literacy and perceived physician characteristics moderate the impact of social processes in OHCs on functional and dysfunctional PERP?

## Methods

### Procedure and Participants

The data for this study came from a Web survey of users of Med.Over.Net (MON), the largest OHC in Slovenia. MON was established in 2000 and offers around 200 online discussion forums. Most of these are moderated by different types of voluntary moderators, among whom are around 150 health-related professionals. In general, the studied OHC covers the following three types of online interactional spaces: (1) online counseling forums in which health professional moderators answer user queries, (2) social support groups forums focused on specific symptoms or health conditions, and (3) general social forums dedicated to topics that are indirectly associated with health issues (parenting, food, relationships, etc). MON registers more than 400,000 visits monthly and has more than 70,000 registered users.

This study was conducted in collaboration with the providers of MON as a part of their annual survey on user experiences and satisfaction with the OHC. The survey, in which respondents participate voluntarily and anonymously, was administered during June 2016 by the OHC provider, who followed ethical standards for administering scientific surveys. The OHC provider invited potential respondents to participate in the Web survey via its email newsletter service. After clicking the link for the Web survey in the email, potential respondents were taken to an informed consent Web page with information about the purpose of research and the length of the survey, an assurance that the data would be dealt with in accordance with national and EU laws, information on who the investigator was, a contact information and a statement that they were under no obligation to participate, and that the aggregated results may be published.

After giving their informed consent and clicking ‘Next’ button respondents could start to fill in the survey. The survey was conducted on platform english.1ka.si, which has mechanisms that disallow multiple entries by the same users. MON is a reputable Web service that treats all personal information (emails) in accordance with national and EU laws and protects data with standard security procedures, which include the deidentification of locally held data files, physical protection of hardware and, strong password protection. The authors of this study had no access to the emails of respondents and received an anonymized dataset containing no identifiable personal information. As per the code of ethics for researchers at the University of Ljubljana [41], no institutional ethics approval was needed for this retrospective type of study. All research was conducted in line with the WMA Declaration of Helsinki on ethical principles for medical research involving human subjects.

The OHC provider first designed a random sample of 40,000 users from the list of all registered users who visited MON at least once in past 6 years. Approximately, 25,000 of these users were randomly assigned to the first Web survey used for this study, whereas the remaining 15,000 users were assigned to a second survey, which mostly focused on service quality and did not provide data for this study. Of approximately 25,000 potential respondents, 4106 (16.42%) clicked the link to the Web survey and 2587 (10.35% participation rate) viewed the informed consent page and clicked the button to start the survey. Of these 1572 finished the survey, which lead to a 60.77% completion rate. The survey took 15 minutes in average. The total response rate of 6.29% (1572/25,000) is small, but not uncommon for Web surveys of this length [42]. The analyses were performed on a subsample of respondents (n=656), who had an encounter with a physician in an ordination at least once in the past 2 years and had visited the forums at MON at least once in the past month. After the exclusion of unit nonresponse, the final sample for analyses contained 591 respondents.

The sample consisted of 16.7% men and 83.3% women (Table 2), whereas the gender structure of the whole portal that hosts the OHC is 70.5% female and 29.5% male, according to Google Analytics. The sample is overrepresented by females, which is likely due to health-related online support groups—which present an important part of the studied OHC—being used predominantly by females, as reported in a review study [43]. Respondents ranged in age from 14 to 74 years (mean 37.6, SD 10.3). More than half (62.9%) of the respondents had at least a college degree, a large majority (74.6%) was married or de facto married and 66.3% were employed or self-employed. In the past 2 years, 36.9% of respondents had visited a physician 7 or more times and 29.9% had had up to 3 visits. In total, 64.0% of respondents reported most often visiting a family or personal physician and 36.0% of respondents reported most often visiting a specialist. Additionally, 41.6% of respondents claimed to have a chronic or acute disease.

**Table 2:** Sample characteristics.

Variable		n (%)
Gender		
	Male	492 (16.7)
	Female	99 (83.3)
Education		
	Lower	45 (7.6)
	Middle	174 (29.5)
	Higher	372 (62.9)
Labor market status		
	School-age youth	53 (9.0)
	Worker, farmer	392 (66.3)
	Retired, unemployed, disabled	131 (22.2)
	Other	15 (2.5)
Marital status		
	Married or de facto married	441 (74.6)
	Single, divorced, widowed	150 (25.4)
Chronic or acute disease		
	Yes	246 (41.6)
	No	345 (58.4)
Physician most visited in past 2 years		
	Family or personal physician	378 (64.0)
	Specialist	213 (36.0)
Frequency of visiting physician in past 2 years		
	Up to 3 visits	177 (29.9)
	3-7 visits	196 (33.2)
	More than 7 visits	218 (36.9)
Total		591 (100)

### Measures

#### Functional and Dysfunctional PERP

The theoretical background for developing items is summarized in Table 1. The initial pool of items was developed by the authors of this study by drawing broadly on two sorts of studies. One on hand, we adopted items from existing measurement instruments that tap aspects of self-efficacy [28,44], competence [3,29], and control [15,29,30,33,45] in patient-physician relationship. On the contrary, several items were newly developed, especially for dysfunctional components of PERP. In developing these items, we strove to achieve high content validity by relying on studies that discuss at least implicitly essential elements of functional and dysfunctional self-efficacy [5,27,34], competence [5,7,15,24,30,31,32], and control [26,27,29,30,40] in relationship with the physician.

Following the standard procedure for scale construction [46], 3 experts (1 in social science methodology, 1 in health communication, 1 one in Internet studies) evaluated an initial pool of 101 items for content validity. On this basis, a refined set of 40 items was selected. These items were put into a survey system and evaluated for clarity, readability, and sensitivity by 5 postgraduate students in Social Informatics trained in survey design and item development. Upon receiving their feedback, we further reduced the item set to 30 altogether. After excluding highly skewed items and items with very low communality (<.2), our exploratory factor analyses on 22 items unveiled 5 latent factors that overlap highly with theoretical components, and in total explain 42.67% of variability in items. Only the dimensions of dysfunctional competences and dysfunctional control were confounded in a single factor. Confirmatory factor analysis demonstrated an acceptable fit for the five-factor model (Root-mean-square error of approximation=.06, standardized root-mean-square residual=.06, Comparative Fit Index=.9). Table 3 presents the factor loadings and Cronbach alphas as measures of reliability. Whereas two dimensions demonstrate satisfactory reliability, the reliability of 3 factors is somewhat below the desired .7. We nevertheless used the scales for further analyses because they are novel and composed of a small number of items, in which case an internal consistency above .6 is also acceptable [47].

**Table 3:** Exploratory factor analysis of the functional and dysfunctional patient empowerment in relationship with physician.

Scale items^a^				Fac 1^b^	Fac 2^c^	Fac 3^d^	Fac 4^e^	Fac 5^f^
I’m prepared for the meeting with my physician so that I get as much as possible out of it.				.70^g^				
When having an encounter with the physician, I make an effort to increase the efficiency of the meeting.				.70				
I am able to attract my physician’s attention if I notice that he/she has become distracted with something else.				.45				
I understand information that I receive from my physician.					.80			
I am able to recollect what my physician said during the encounter.					.87			
I can describe my symptoms to the physician in a very clear way.					.40			
Occasionally I have trouble understanding my physician’s instructions (reversed item)					-.54			
I have the confidence to express possible doubts about the therapy that a physician would recommend						.41		
I do not need to just listen to the doctor; I can also suggest something.						.51		
If I see or read important health-related information, I usually mention it to the physician.						.40		
Even though I do not read medical journals, I believe that I have more knowledge about my health problems than my physician does.							.70	
I like to provoke the physician with information that he/she may not be aware of.							.50	
I diagnose my condition with the help of the Internet, and I go to the physician just to get his/her confirmation of my diagnosis.							.51	
I do not need to ask my physician about instructions for medication or therapy because I am more knowledgable about this than he/she.							.62	
I would rather not ask questions of my physician because I am sure to find better explanations elsewhere (like on the Internet, from my friends, etc)							.66	
Sometimes I do not listen to my physician because I know in advance what he/she is about to say.							.67	
Sometimes I disagree with my physician just to show him/her that he/she is not always right.							.52	
I began to visit my physician more frequently just to complain about his/her previous procedures.							.43	
I know that I could ask my physician more questions, but I probably would not get any useful advice.							.52	
I can convince my physician to approve a longer sick leave for me if necessary.								.70
A physician could not stop me from getting medicine if I really wanted to get it.								.45
I can persuade physician for appointment with (another) specialist even if not needed								.63
Cronbach alpha				.66	.76	.66	.81	.64
					
					

^a^All items are on scale ranging from 1-completely disagree to 5-completely agree.

^b^Fac 1 corresponds to the factor functional self-efficacy.

^c^Fac 2 corresponds to functional competences.

^d^Fac 3 corresponds to functional control.

^e^Fac 4 corresponds to dysfunctional competences and control.

^f^Fac 5 corresponds to dysfunctional self-efficacy.

^g^Only factor weights of absolute value equal or larger than .40 are reported.

#### Social Processes in OHCs

Items were developed on the basis of an empowerment processes scale [10,13] which delineates the main social processes that are important for patient empowerment. The original scale with 29 items measuring 5 dimensions (exchanging informational support, receiving emotional support, giving support, self-expressing, and finding meaning) was supplemented with a measure of information exchange with health professional moderators in the OHC. We identified the latter dimension as OHCs are increasingly involving online health professionals, who might strengthen and qualitatively enrich OHCs’ informational support as they filter health-related information against scientific knowledge and thus improve the reliability and objectivity of experiential knowledge in OHCs [8,48,49]. The reliability of the scale was satisfactory because the Cronbach alphas ranged from .80 to .91.

#### The eHealth Literacy Measure

This eHealth Literacy measure was adopted from the eHEALS Scale [50]. As Norman [51] identified several issues with the eHEALS Scale, we slightly modified some items and reversed a few of them to make the scale less prone to social desirability. The scale failed to demonstrate unidimensionality, but for the analysis we decided to retain a five-item factor, which seemed most representative of the eHealth literacy construct (example item: “I have difficulties separating quality health information from less-quality ones on the Internet” [reversed item]). The scale was on the margin of acceptability (.71).

#### The Perceived Paternalism of Physician Measure

The perceived paternalism of physician measure was adapted from the STAR Scale [52], which relates to what extent a physician is perceived to be paternalistic or cooperative. A five-item scale (example item: “My physician does not allow me to express my thoughts and opinions”) demonstrated satisfactory reliability (.84). Higher values represent a more paternalistic physician, whereas lower values represent a more cooperative one.

### Analyses

A series of exploratory factor analyses were conducted to explore the factor structure of the scale to measure functional and dysfunctional PERP and to determine what items of the scale should be retained. Factors were extracted using Principal Axis Factoring with oblimin rotation as we didn’t expect orthogonal factor solution. The number of factors was selected on the basis of eigenvalues higher than 1. This decision was also supported by inspection of the scree plot. The obtained factor solution was put into a confirmatory factor procedure, which resulted in several statistics that estimate goodness of fit of the factor model to the study data. Since the statistics showed good fit of the model, no modifications were needed.

To analyze RQ_1_ and RQ_2_, we conducted a multiple regression analysis. As our factor analysis revealed 5 dimensions (factors) of dependent variable (PERP), a regression analysis had to be conducted for each dimension separately. More precisely, a hierarchical ordinary least squares multiple regression analysis approach [53] was used. This was conducted in such a way that 3 successive linear regression models were estimated for each of the 5 PERP dimensions. In step one, a model with only control variables (sociodemographics, length of relationship with physician, presence of acute or chronic disease) was estimated. In step two, independent variables (social processes in OHCs) were added, and in step three, two moderating variables (eHealth literacy, perceived physician characteristics) and the interactions between them and social processes in OHCs were added. This procedure allows researcher to test if successive model fits better than the previous one. The comparison of models in step two and models in the step one provided insight into RQ_1_, whereas comparison of the models in step three and the models in step two allowed us to analyze RQ_2_. All variables that appear in interactions were a priori centered to avoid collinearity. A logarithm of a scale for dysfunctional competences and self-efficacy was used because the original variables were highly skewed.

## Results

### Descriptive Statistics of Variables in the Model

Among the 5 dimensions of the PERP, the predominant one was functional competences (mean 4.09, SD 0.57). We also noticed an above-average presence of functional self-efficacy (mean 3.96, SD 0.65) and functional control (mean 3.49, SD 0.71) in relationships with the physician. All components of dysfunctional PERP were present to a lesser extent. Comparatively, the one predominantly present was dysfunctional self-efficacy (mean 2.39, SD 0.81), followed by dysfunctional competences and control (mean 1.97, SD 0.65). The latter variable was highly skewed because only 5% of all respondents on average responded to items with agree or completely agree values, which shows that only a small proportion of respondents obtained power by taking an explicitly manipulative, disrespectful, and generally negative stance toward the physician.

Among the social processes, the predominant (Table 4) in the studied OHC was exchanging information with health professional moderators (mean 3.66, SD 0.73), followed by exchanging information with users (mean 3.17, SD 0.67). The processes of receiving social support (mean 2.84, SD 0.93) and sharing experiences (mean 2.62, SD 1.1.8) were present to a lesser extent. Giving social support was a rare practice among the studied sample (mean 1.91, SD 0.91). Descriptive analyses of moderating variables showed that eHealth literacy was quite dispersed among the sample, with the majority being in the middle range (mean 3.31, SD 1.86). Users also reported that their physicians were, on average, more cooperative than paternalistic (mean 2.34, SD 0.88).

**Table 4:** Descriptive statistics of dependent, independent, and moderating variables in the model.

Type	Variable	Mean (SD)	Min	Max
Dependent	Functional self-efficacy	3.96 (0.65)	1.3	5
	Functional competences	4.09 (0.57)	2.2	5
	Functional control	3.49 (0.71)	1	5
	Dysfunctional self-efficacy	2.39 (0.81)	1	5
	Dysfunctional competence/control	1.97 (0.65)	1	5
Independent	Exchanging information with users	3.17 (0.67)	1	5
	Exchanging information with health professional moderators	3.66 (0.73)	1	5
	Receiving social support	2.84 (0.93)	1	5
	Giving social support	1.91 (0.91)	1	5
	Sharing experiences	2.62 (1.18)	1	5
	Finding meaning	2.84 (0.90)	1	5
Moderating	eHealth literacy	3.31 (1.86)	0	6
	Physician's paternalism	2.34 (0.88)	1	5

### Analysis of Research Questions

Five hierarchical regression analyses were conducted for each one of 5 dependent variables that correspond to the 5 dimensions of PERP. Results of hierarchical regression analyses, where functional PERP is the dependent variable, are reported in Table 5; whereas results of hierarchical regression analyses that pertain to dysfunctional PERP as dependent variable are reported in Table 6. To illustrate an example of regression on functional self-efficacy in relationship with the physician (third column in Table 5), we first entered control variables as predictors in the model in step one. This model did not fit to the data (R^2^_adj_=.002, *P*=.78). In step two, predictors that pertain to social processes in OHC were entered and this model significantly fit the data (R^2^_adj_=.05, *P*<.001). Moreover, in comparison with the model in step one, the increase in R^2^ was also significant (note that in SPSS, the difference in R^2^ is reported and not the difference in R^2^_adj_), thus suggesting that the model in step two is more valid for interpretation. In step three, moderating variables were entered together with interactions with predictors in step two. A significant increase in R^2^ (ΔR^2^=.116, *P*<.001) was noted, making the model in step three the most valid for interpretation. Such a hierarchical regression analysis approach was repeated for the other 4 dimensions of PERP, and the results consistently showed that the best-fitting models were those in step three (R^2^ adjusted, ranging from .19 to .28), except for the model on dysfunctional self-efficacy where the fit was nonsignificant (R^2^_adj_=.01, *P*=.61). We nevertheless considered regression coefficients for interpretation.

For a detailed analysis of RQ_1_, the regression coefficients of the predictors of the model in step two needed to be investigated. Among all social processes on OHC, exchanging information with health professional moderators proved to be the most important factor of PERP. It had a weak but statistically significant impact on functional self-efficacy (beta=.12, *P*=.02) and functional control (beta=.16, *P*=.005), and negative impact on dysfunctional competences and control (beta=−.12, *P*=.03), thus enabling a more cooperative relationship with the physician. Exchanging information with users also proved to be a predictor of functional self-efficacy (beta =.12, *P*=.05) but had no impact on the other two components of functional empowerment. In addition, it demonstrated a positive, if marginally significant, impact on dysfunctional competences (beta=.11, *P*=.08). Significant predictors of functional self-efficacy proved to be self-expressing (beta=.13, *P*=.01) and giving social support (beta=.13, *P*=.02). Self-expressing also had marginally significant impact on competences (beta =.09, *P*=.08) and a negative impact on dysfunctional self-efficacy (beta=−.12, *P*=.05). Interestingly, receiving social support had a significant but negative influence on functional self-efficacy (beta=−.21, *P*<.001), whereas it had a positive impact on dysfunctional competences (beta=.11, *P*=.05).

**Table 5:** Regression coefficients of independent, contextual, and control variables and interactions on dimensions of functional patient empowerment in relationship with the physician (PERP).

	Predictor	Self-efficacy		Competence		Control	
		beta	*P* value	beta	*P* value	beta	*P* value
**Step 1**							
	Gender	.06	.17	.02	.65	.03	.58
	Age	.06	.17	.10	.03	.08	.09
	Education	.02	.65	.09	.07	.03	.55
	Chronic or acute disease (0=no,1=yes)	−.04	.38	−.02	.62	−.09	.07	
	Length of relationship with physician	−.04	.42	.06	.20	.03	.55
**Step 2**							
	Exchanging information with users	.12	.05	.01	.85	.09	.10
	Exchanging information with HPM^b^	.12	.02	.05	.36	.16	.005
	Receiving social support	−.21	<.001	−.08	.12	−.09	.13	
	Giving social support	.13	.02	−.02	.70	.10	.07
	Finding meaning	.02	.77	−.01	.88	−.06	.37
	Self-expressing	.13	.01	.09	.08	.05	.38
**Step 3**							
	Physician’s paternalism	−.23	<.001	−.25	<.001	−.24	<.001	
	eHealth literacy	.18	<.001	.34	<.001	.23	<.001
	Finding meaning X^c^ Physician’s paternalism^a^			−.12	.06		
	Exchange info with HPM X eHealth literacy^a^					.14	.01
	Finding meaning X eHealth literacy^a^						
	Receiving social support X Physician’s paternalism^a^							
R^2^_adj_(step 1)		.002	.46	.027	<.001	.010	.20
R^2^_adj_ (step 2)		.050	<.001	.050	<.001	.051	<.001
ΔR^2^		.053	<.001	.035	<.001	.054	<.001
R^2^_adj_ (step 3)		.200	<.001	.279	<.001	.193	<.001
ΔR^2^		.116	<.001	.244	<.001	.161	<.001

^a^Only significant interactions are reported.

^b^HPM: health professional moderators.

^c^X denotes interaction between two variables.

**Table 6:** Regression coefficients of independent, contextual, and control variables and interactions on dimensions of dysfunctional patient empowerment in relationship with the physician (PERP).

	Predictor	Self-efficacy		Competence and Control	
		beta	*P* value	beta	*P* value
**Step 1**					
	Gender	−.03	.59	.02	.74
	Age	.02	.84	−.06	.23
	Education	−.01	.83	.01	.92
	Chronic or acute disease (0=no,1=yes)	−.01	.80	−.02	.64
	Length of relationship with physician	−.02	.86	−.02	.68
**Step 2**					
	Exchanging information with users	.03	.67	.11	.08
	Exchanging information with HPM^b^	−.02	.78	−.12	.03	
	Receiving social support	.02	.81	.11	.05
	Giving social support	.01	.89	.07	.19
	Finding meaning	.05	.52	.02	.70
	Self-expressing	−.12	.05	−.08	.15
**Step 3**					
	Physician’s paternalism	.01	.79	.40	<.001	
	eHealth literacy	.08	.27	.06	.42
	Finding meaning X^c^ Physician’s paternalism^a^			.14	.03
	Exchange info with HPM X eHealth literacy^a^	.10	.09		
	Finding meaning X eHealth literacy^a^			−.17	.01
’	Receiving social support X Physician’s paternalism^a^			−.11	.08	
R^2^_adj_(step 1)		.000	.70	.000	.93
R^2^_adj_ (step 2)		.000	.81	.041	.001
ΔR^2^		.015	.93	.058	<.001
R^2^_adj_ (step 3)		.010	.61	.218	<.001
ΔR^2^		.037	.10	.194	<.001

^a^Only significant interactions are reported.

^b^HPM: health professional moderators.

^c^X denotes interaction between two variables.

For a detailed analysis of RQ_2_, the regression coefficients of the predictors that were entered in the model in step three needed to be investigated. In all models, except for dysfunctional self-efficacy, the perceived paternalism of the physician had a moderate influence on the functional components of empowerment (beta=−.23 to −.25, *P*<.001) and a strong impact on dysfunctional competences and control (beta=.4, *P*<.001). These estimates showed that the more physicians were perceived to be paternalistic, the less the patients were able to develop functional empowerment and the more they developed dysfunctional competences and control in relation to physicians. The eHealth literacy, as another moderating variable, demonstrated a significant impact on all 3 components of functional empowerment (beta=.18 to .34, *P*<.001) but not on the dysfunctional ones.

Table 5 also reports interactions between eHealth literacy and social processes in OHC and between the perceived physician characteristics and the social processes in OHC. We noted that respondents who found meaning under the condition of a paternalistic physician were less likely to exhibit functional competences in their relationships (beta=−.12, *P*=.06). Conversely, this condition likely leads to the development of dysfunctional competences and control (beta=.14, *P*=.03). High eHealth literacy intensified the effect of exchanging information with health professional moderators on functional control (beta=.14, *P*=.01), but it surprisingly also led to more dysfunctional self-efficacy (beta=.10, *P*=.09). People who found meaning in the OHC and had high eHealth literacy were less likely to develop dysfunctional competences and control (beta=-.17, *P*=.01). Finally, receiving emotional support under the conditions of a paternalistic physician were less likely to lead to the development of dysfunctional competences and control (beta=−.11, *P*=.08).

## Discussion

### Emergence of Dysfuntional Empowerment

The main goal of this research was to investigate whether social processes in which users are involved in OHCs have any impact on the patient empowerment experienced in relationships with the physician and, if so, what is the nature of such an impact. Beginning from the limitations of previous studies, we conceptualized the PERP and on this basis we developed a relatively valid and reliable scale. As this is a proposal of a new scale, it has some issues with reliability. Nonetheless, it demonstrates a meaningful factor structure, indicating that PERP should be investigated along at least two dividing lines: (1) two types of communicative orientation of a patient toward the physician and the associated functional or dysfunctional outcomes for the patient-physician relationship and (2) 3 components of patient empowerment in relationship with the physician: self-efficacy, control, and competence. Such conceptualization might be useful for further research on the dynamics of patient-physician relationships in the online era.

To claim that empowered patients are emerging solely due to processes in OHCs would be a gross overstatement, as the results clearly show that it is foremost patient’s eHealth literacy and physician’s personality and communication style that are driving PERP. However, even after controlling for these 2 determinants, we could show that some social processes in which users of OHCs are involved do have an impact on how patients relate to their physicians.

### Getting Clinical and Experiential Knowledge as an Empowering Process

The most important factor pertaining to activities in the OHC was the involvement in exchanging information with health professional moderators. This influenced patients to (1) become more efficient in their relationships with the physician, so that they knew what to ask and were motivated to have collaborative and efficient meetings and (2) get more control in terms of having confidence to ask questions and participate in decisions. The impact on control was attenuated under the condition of high eHealth literacy. Furthermore, respondents exchanging information with health professional moderators were less likely to develop overconfident and strategically self-interested stances toward their physicians. The results reveal, however, that exchanging information with health professional moderators for those with high eHealth literacy can be somewhat problematic. People who know a lot and talk a lot with experts in OHCs might learn communication skills [8] to strategically deal with their physician in order to efficiently strive for their own private, often non-legitimate interests, such as getting medicine no matter what and simulating disease, as manifested by a raised dysfunctional self-efficacy.

Exchanging experiential information with users had a conflicting impact on PERP. On one hand, it had a positive impact on self-efficacy, suggesting that interaction with other users helps develop skills for more efficient communication with physicians. However, it can also lead to overconfidence in one’s own competences in relation to the professional competences of the physician, as already suggested in previous research [17,20]. The concerns with accuracy and completeness of information in peer exchanges in OHCs have already been raised in previous studies, but we can only conclude, as does Sillence [54], that the exchange of experiential information is a complex process that does not have a simple unidirectional impact on relationship with the physician.

### Problematic Facets of Exchanging Social Support

Our results revealed a surprising finding about the role of receiving social support, something often hailed as one of the most important processes in OHCs in terms of leading to individual empowerment [7,15]. In particular, people who are getting advice, consolation, and other types of emotional support from other users likely report less self-efficacy in relationships with their physicians and more dysfunctional competences. Why would people with support in OHCs become less self-efficient in relationships with their physicians, undermining a chance for meaningful and efficient encounters? And why would people who get support from other users in OHCs be more inclined to become overconfident in relation to their physician, start to distrust them, and develop feelings of superiority over professional knowledge? The regression analysis did not give answers to these questions, but these associations could be attributed to the plausible prevalence of “nurturant” social support [55], which is fitted to people who do not search for true causes of their disease but rather for ways to manage negative feelings and emotions in a short-term manner [56]. In this process, they blame external factors (also physicians) for their inability to solve their health problems [56]. Receiving social support in OHCs can also form expectations of physicians providing support [57] and as the latter are perceived to be too busy for supportive discussions [58], it may be that the patients in this process also develop an aversion toward the physician. This way they become less efficient in relation with the physician and develop uncooperative and generally negative attitudes toward the physician and the professional knowledge he or she represents.

Receiving emotional support does not have negative impacts in all cases. The problematic effects of social support are diminished if a patient’s physician is perceived as paternalistic, since social support in OHCs in this case lowers dysfunctional competences and control. This might be connected to the finding that the more the patients exchange social support in OHCs, the less likely it is that they will discuss information found online with the health professionals [4]. We could speculate that those patients whose physicians are perceived to be paternalistic and supposedly unsupportive probably satisfy their need for social support in OHCs, consequently diminishing their expectation of getting social support from their physicians. Relieved of the expectation of a supportive physician, a patient is less likely to develop a negative attitude toward the physician.

Although receiving social support seems to have an ambivalent role in the PERP, giving social support demonstrated a positive impact. Providing other users with help and advice bestowed patient’s confidence and competence in asking the right questions to physicians and making meetings more efficient. Those who gave support to other OHC users were also more likely to engage in shared decision making with their physicians. This is in line with the general finding from online community research that people who are active contributors in online environments are usually also more proactive in their offline environment [59]. Writing posts for the purpose of self-expression also had a positive impact on the functional components of PERP, which confirms the results of previous studies [4,15,16]. Self-expression stimulates cooperative self-efficacy, which supports findings that writing stories and expressing disease in safe “testing” environments has positive impacts on a patient’s competence and efficiency [7], as well as on the level of his or her relationship with the physician.

### Does Finding Meaning Diminish Meaning in Interactions With the Physician?

Patients who found meaning in their life and made sense of their situation with the help of other users and who at the same time perceived their physicians as paternalistic through OHC were less likely to develop competences for more understanding relationships with the physician. As such, they will be more likely to develop dysfunctional competences and control, opening possible conflicts between the 2 partners. One possible explanation for this result might lie in the finding from previous studies that information received from external resources like OHC is often perceived to be superior to that provided by the medical staff [17]. This connects to findings that complementary and alternative medicine fills the gaps that evidence-based medicine leaves behind in terms of providing a more holistic, therapeutic approach with treatments that are easier to understand and follow [60]. Moreover, as exchanges of advice in OHCs are often based on similar beliefs [48] that can be based on opposition to evidence-based medicine [36], it is plausible that users find new meanings and purposes that involve a negative and underestimating attitude toward their physicians.

Finding meaning can on the other hand be a positive empowerment process, as under condition of high eHealth literacy, finding meaning had an inhibiting effect on dysfunctional competences and control. More precisely, people who are digitally literate in health issues and use OHCs to find meaning in exchanges with other users will be less likely to develop a problematic stance toward their physicians.

Although not a central focus, it needs to be pointed out that this study confirms an important role of eHealth literacy in the dynamics of patient-physician relationships [61]. People who successfully encompass a constellation of digital literacy, health literacy, media literacy, and other literacies [50] will also develop competences and skills for a successful relationship with the physician, which enables better understanding and retention of information and improved ability to ask meaningful questions. Furthermore, this characteristic will also allow patients to be more effective in their relationships with their physicians and have more control in terms of participation in shared decision making.

### Limitations

This study has some methodological limitations that warrant further research. First, because the respondents in this study were recruited from a single OHC, generalizations of any findings should be made with caution because OHCs can vary greatly in their membership, focus, and role structure [62]. Ideally, the research should be repeated in various national contexts and with different types of OHCs, focusing on different diseases and user needs. Second, although the proposed measure of PERP presents an improvement over single-item measures, the scale is novel and consequently did not go through enough testing, thus rendering it marginally valid and reliable. However, we hope that researchers will recognize its value and work further to improve its psychometric qualities. In connection arises a third limitation that the patient’s perception of empowerment in the relationship with the physician is only one part of a dyad, or even triad [39]. To observe the relationship completely, the communication orientation of physicians should be considered, but this would demand a much more complex research design.

Finally, in this study, we proposed a theoretical model in which social processes in OHCs have an impact on PERP, but it needs to be stated that, empirically, the association between variables can also go in the opposite direction. Moreover, the 2 phenomena are probably in a recursive relationship, where processes in OHCs are influencing relationships with the physicians and patients’ experiences with physicians can impact how they use OHCs and other online tools. To address this issue, we may have to undertake a random control trial type of research design.

### Practical Implications

On the basis of these results, several important messages can be discerned for OHC managers, users, patients, and health care practitioners. First, strong empirical evidence for the importance of health professionals being visibly present in OHCs should stimulate online community managers to invest energy in attracting physicians and other health experts to actively participate and moderate discussions in OHCs. They are not only carers of high-quality information in OHCs, but they also stimulate processes that can ease patients’ relationships with their physicians (whether family doctors or specialists) [8]. Second, both patients and physicians should be aware of ambiguous processes in OHCs and their impact not only on patients handling their diseases but also on the fact that patients encounter health experts in different venues—physically in ordination and online in OHCs and other platforms. Physicians should be aware of the processes in OHCs and other experiences in which patients are involved, providing material for arriving at decisions that would lead to better health outcomes. If all these encounters are not discussed, conditions might form that open space for conflicts and strategic action, which in the end is harmful for the patient and physician.

We believe that a better understanding of such aspects of patient-physician relationships is required because it has implications for how physicians are trained and health care is organized. We know that if physicians surrender their control, they can create an atmosphere of respect that facilitates the positive aspects of empowerment [9]. Now we also know that if physicians do not surrender their paternalistic role, empowerment can go astray into its dysfunctional mode. Finally, it seems that although OHCs are environments where patients can get emotional support, this does not mean that patients should not expect supportive communication from their physicians. Our results suggest that some patients prefer what we call passive support, which is composed of compassion and consolation and does not imply any action on the patient’s part. However, we could speculate that physicians are more inclined to give patients something we call active support—a sort of compassion but one with proactive components—for which the patient is advised to more actively take care of his or her health. Some patients see these two supports as conflicting, and it might be advisable for OHC managers to emphasize such proactive support so that patients do not fall into a bubble where exclusively external factors are blamed for their current health statuses.

### Conclusions

To our knowledge, this is the first study with a quantitative approach to empirically demonstrate the problematic aspects of patient empowerment that should be considered when investigating the impact of a patient’s engagement in online environments, both as an information seeker and as an active coproducer of online environments. Patients can feel psychologically empowered, and this sort of acquired internal power can translate into a cooperative relationship with the physician, thus bringing positive outcomes for both. We termed such empowerment functional. However, a patient’s feelings of empowerment can also be transformed, especially under conditions of low eHealth literacy and the presence of a paternalistic physician, into a manipulative, disrespectful, and generally negative stance toward the physician. Such empowerment is not productive for the relationship and possibly leads to conflicts as well as worsens the health outlooks for the patient.

Online environments can have many benefits for patients, but unchecked information, under conditions of poor literacy, can lead to social contagion processes [63] that can have dangerous implications for patients and for wider societal processes [64]. Once we are aware of such processes, we can start addressing them. Our findings suggest that they can be inhibited by filtering experiential knowledge through health professional moderators and through raising awareness regarding the importance of eHealth literacy when dealing with online health information. In addition, to achieve true empowerment, this concept should not be understood solely from the perspective of an individual benefits. It also needs to include a perspective of working together for our shared interests and to improve our interactions, communities, and institutions [65].

## Abbreviations

MON: Med.Over.Net
OHC: online health community
PERP: patient empowerment in relationship with physician
